# Influence of daily life and health profile in subtle cognitive decline of women residing in Spanish religious communities: DeCo religious orders study

**DOI:** 10.3389/fpubh.2024.1395877

**Published:** 2024-07-17

**Authors:** Teresa Lopez de Coca, Lucrecia Moreno, Juan Pardo, Jordi Pérez-Tur, Hernán Ramos, Victoria Villagrasa

**Affiliations:** ^1^Cátedra DeCo MICOF-CEU UCH, Universidad Cardenal Herrera-CEU, CEU Universities, Valencia, Spain; ^2^Department of Pharmacy, Universidad Cardenal Herrera-CEU, CEU Universities, Valencia, Spain; ^3^Embedded Systems and Artificial Intelligence Group, Universidad Cardenal Herrera-CEU, CEU Universities, Valencia, Spain; ^4^Institut de Biomedicina de València-CSIC, CIBERNED, ISCIII, Valencia, Spain

**Keywords:** cognitive dysfunction, dementia, depression, nun study, screening

## Abstract

**Background:**

Prior to the onset of dementia, individuals commonly undergo a phase marked by subtle cognitive changes, known as subtle cognitive decline. Recognizing these early cognitive alterations is crucial, as they can serve as indicators of an impending decline in cognitive function, warranting timely intervention and support.

**Objectives:**

To determine the incidence of subtle cognitive decline in a population of Spanish women and establish the relationship with possible protective and/or risk factors such as cognitive reserve, cardiovascular risk factors, medication consumption and psychosocial factors.

**Design and participants:**

This is a cross-sectional observational study with women from the general population and a more homogeneous population composed of nuns from the Valencian region (Spain).

**Measurements:**

A validated questionnaire was used including lifestyle variables, chronic illnesses, level of education and pharmacological treatments. Three validated subtle cognitive decline screening tests with varying levels of sensitivity and specificity were used: Memory Impairment Screening, Pfeiffer’s Short Portable Mental State Questionnaire, and Semantic Verbal Fluency.

**Results:**

Our results suggest that nuns may have a significantly reduced risk of cognitive decline compared to the general population (20.67% in nuns vs. 36.63% in the general population). This lower risk for subtle cognitive decline in nuns may be partly attributed to their higher cognitive reserve and long-time engagement in intellectually stimulating activities. Additionally, nuns tend to adopt healthy lifestyles, they are not isolated because they live in community and obtained lower scores for risk factors such as depression, anticholinergic burden, and benzodiazepine consumption.

**Conclusion:**

A healthy lifestyle combined with intellectually stimulating activities is related with preserved cognitive function.

## 1 Introduction

Dementia constitutes a clinical diagnosis characterized by cognitive symptoms that substantially impede an individual’s ability to engage in daily activities. This condition is typically heralded by a phase of subtle cognitive changes known as Subtle Cognitive Decline (SCD), often discernible in individuals testing positive for Alzheimer’s disease biomarkers (1–3). Although a definitive treatment for dementia is presently unavailable, the timely administration of neuroprotective drugs before the onset of the disease becomes crucial to potentially mitigate ensuing damage. Therefore, early detection of SCD is paramount to identifying candidates for clinical trials aimed at modifying disease progression (4, 5). Contemporary clinical research is oriented towards the early diagnosis of the transitional phase between normal aging, SCD, and dementia (6). The main risk factors for ‘sporadic’ late-onset Alzheimer’s disease and other related dementias include age, family history and genetic susceptibility, with the ε4 allele of apolipoprotein E being a notably genetic risk factor. Unfortunately, none of these risk factors can be modified by medical intervention or individual behavior (7). Nonetheless, there exist other modifiable risk and protective factors (3). Thus, to effectively mitigate the impact of degenerative processes, it is imperative to develop a comprehensive methodology for identifying individuals in the early stages of cognitive problem development. Achieving this objective will facilitate the identification and management of environmental and lifestyle risk factors contributing to the progression of these conditions.

**Figure 1:**
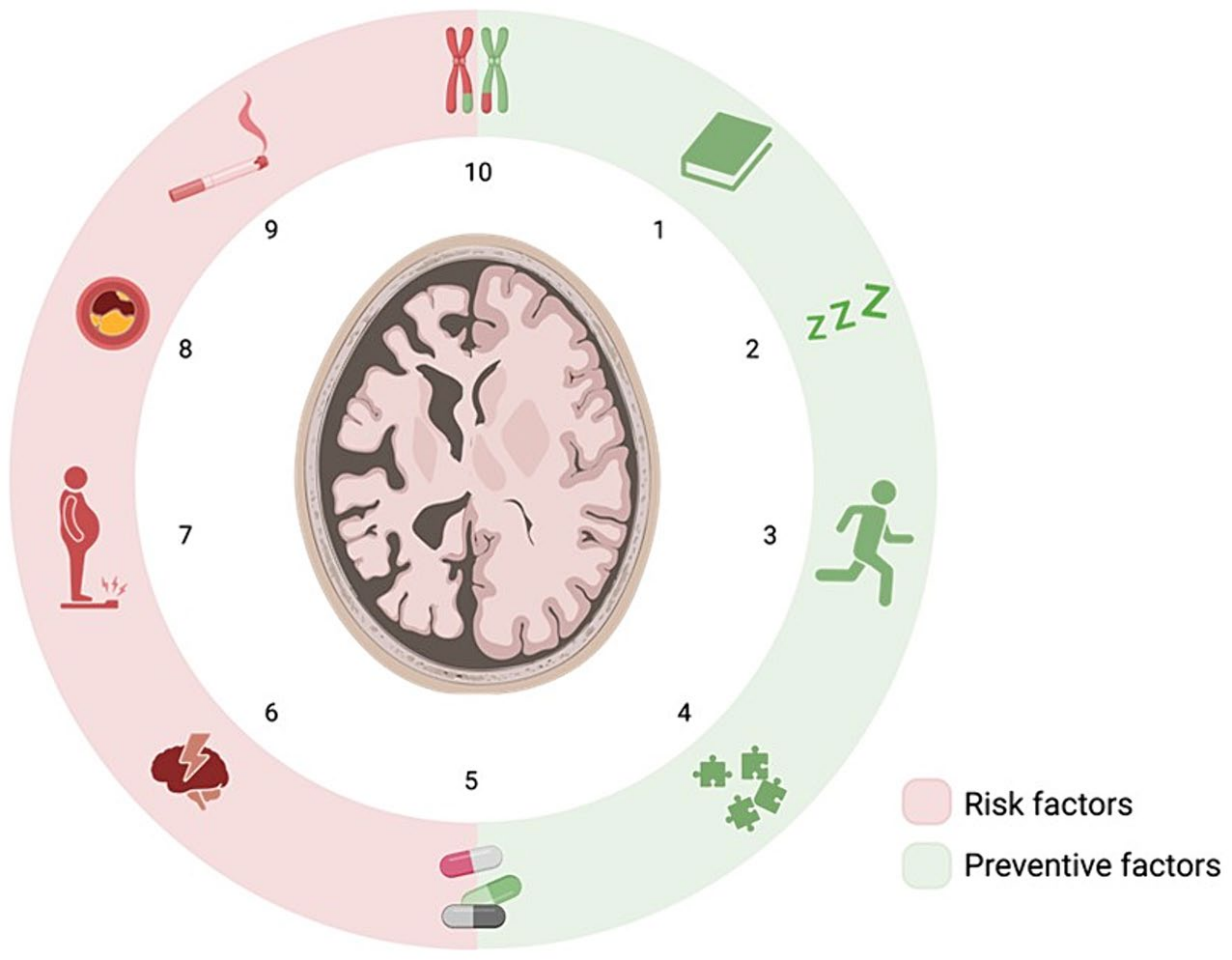
Risk and preventive factors for cognitive impairment. Preventive factors include reading (1), sleep (2), physical activity (3), and cognitive stimulation game (4). Risk factors include depression (6), overweight (7), atherosclerosis (8) and smoking (9). Also, genetics (10) and medication (5) play a dual role.

Several factors, including those associated with cognitive reserve (such as reading, engaging in studies, playing board games, etc.) and cardiovascular risk (CVR), among others, are considered modifiable (3). Aspects such as educational level, stimulating occupations, internet usage, and reading have been identified as protective factors for mental health. Over the course of our lives, cognitive stimulation contributes to enhanced cognitive development, resulting in a notable reduction in the frequency and severity of behavioral and psychological symptoms (8). On the other hand, CVR encompasses factors such as smoking, arterial hypertension (AHT), body mass index (BMI), or diabetes mellitus (DM), which pose risk to mental health. Psychosocial factors like depression, use of drugs with anticholinergic effects or subjective memory complaints (SMC), which is characterized by self-perceived memory impairment, are also contribute to these risk factors. Previous studies have shown that the prevalence of obtaining evidence consistent with SCD is higher in patients with SMC (9–12). In addition, antagonizing with muscarinic receptors due to the use of drugs with anticholinergic effects can lead to cell death and SCD. This leads to a direct link between SCD and use of anticholinergic drugs (13). Figure 1 shows the influence of these factors listed above.

A significant limitation in studies focused on identifying markers of early cognitive decline (14) lies in the potential influence of various external factors, primarily those related to lifestyle. One approach to mitigate this limitation involves working with cohorts of individuals exposed to the same environment for extended periods, thereby minimizing the impact of diverse exogenous factors. An exemplary instance of this methodology is the examination of individuals residing in religious orders, where the consistent environment offers a more controlled setting for investigating cognitive changes. Individuals in religious orders maintain homogeneous and health-conscious lifestyles: they adhere to nearly identical dietary patterns, follow similar daily schedules, receive uniform medical care, exhibit a high level of discipline, and the majority abstain from smoking or alcohol consumption (15).

The initial community-based studies encompassed the Nun Study, the Honolulu Aging Study (United States), and the Hisayama Study (Asia) (15–17). Subsequently, the Religious Order Studies (ROS) and the Rush Memory and Aging Project (MAP) were initiated, both of which originated in the United States. These investigations were primarily directed towards identifying sensitive and specific mechanisms for disease detection, rather than emphasizing prevention through the modification of certain risk factors (12). Notably, no European nun studies have been published, and there is a dearth of studies minimally comparable to those conducted on the Spanish population. In Spain, where cardiovascular risk is low, and adherence to a Mediterranean diet is prevalent, such research has yet to be undertaken.

Hence, the objective of the current study is to ascertain the incidence of Subtle Cognitive Decline and to establish its correlation with potential protective and/or risk factors. Specifically, the investigation will explore associations with cognitive reserve, cardiovascular risk factors (CVR), psychosocial and lifestyle factors within a homogenous population of nuns in the Valencian region of Spain.

## 2 Methods

### 2.1 Study design

This cross-sectional observational study aims to evaluate risk and/or prevention factors associated with SCD in a population of nuns, comparing them with women recruited from community pharmacies. Both study groups reside in the Valencian region (Spain). The research adhered to the guidelines stipulated by the European General Data Protection Regulation (GDPR) and the Spanish Organic Law 3/2018 on the Protection of Personal Data and the Guarantee of Digital Rights. Furthermore, the study adhered to the ethical principles outlined in the Declaration of Helsinki. All participants provided written informed consent, in accordance with the requirements of the European GDPR. The study protocol underwent a thorough review and received approval from the Institutional Review Board (IRB) at the CEU Cardenal Herrera University (CEEI21/201, approval date: 16 July 2021).

### 2.2 Study design and participants

The study was conducted from September 2021 to November 2022. Participant recruitment involved direct engagement with the Abbesses of each religious order. The Mother superior, in turn, provided each potential participant with general information about the study in order to recruit volunteers. Subsequently, research team members conducted interviews at the residence of each congregation. The control group consisted of women from the general population (WGP) recruited from 19 community pharmacies in the Valencian region of Spain (18).

#### 2.2.1 Inclusion criteria

The only inclusion criteria was to be at least 50 years of age. Each recruited nun was subsequently matched with a woman from the general population based on age.

#### 2.2.2 Exclusion criteria

The exclusion criteria encompassed individuals with any diagnosis of dementia, intellectual disabilities, and/or those experiencing severe sensory deficits (such as blindness or deafness) or physical incapacity that hindered the ability to participate in interviews.

### 2.3 Sample size

We used the G*Power statistical program to study the sample size necessary for this analysis (19). A two-sample *t*-test was performed to check statistical differences between two independent means (two groups). The parameters were established as a two-sided test with a medium effect size (0.5), a power of 0.95, and a significance level of 0.05. This concluded with a minimum sample size of 105 subjects for each group.

### 2.4 Interview procedure

The study was explained to the participant, followed by a personal interview lasting approximately 1 h. During this session, anthropometric measurements were obtained, and Subtle Cognitive Decline was assessed using 3 validated tests: (a) Memory Impairment Screen (MIS), (b) Short Portable Mental Questionnaire (SPMSQ) and (c) Semantic Verbal Fluency Test (SVF). The selection of these tests was based on the recommendations of the Valencian Society of Neurology.

a) The MIS evaluates verbal learning through reading, followed by free and facilitated recall of four words. The MIS demonstrates a strong correlation with measures of hippocampal and entorhinal volumetry. The Spanish MIS adaptation followed the same criteria as the original, using well-known categories and medium prototypicality words to avoid random answers. Patients read and identified four words from different categories, then recalled them after an interference task. If they missed any words, category cues helped with recall. Scoring: total = (free recall × 2) + (facilitated recall x 1) (20–23). Scoring is on a scale ranging from 0 to 8, and the established cut-off point indicative of potential Subtle Cognitive Decline (SCD) is less than or equal to 4 points. In the Spanish population, the MIS has shown a sensitivity of 80% for dementia, coupled with a specificity of 96% (20–23).
b) The SPMSQ (24) assesses different intellectual aspects, encompassing short-term memory, long-term memory, orientation to surroundings, knowledge of recent events, and the ability to perform sequential mathematical tasks. The Spanish version of SPMSQ, is considered valid for the illiterate population due to its simplicity and is extensively employed in primary care. Characterized by brevity and portability, the test items provide valid information to assess orientation, memory function related to self-care, remote memory, and the ability to perform serial mental operations. Each subject must answer all 10 questions without memory aids like calendars or newspapers. Refusal to answer typically indicates lack of knowledge and should be scored as incorrect, especially if the subject has been cooperative up to that point (25). A determined cut-off point indicates possible SCD with the presence of 3 or more errors. The Spanish version of this test exhibited a sensitivity of 85.7% and a specificity of 79.3%, respectively.
c) The VSF is extensively utilized in neuropsychological assessments, recognized for its ease of use and expediency. It proves highly sensitive and specific, enabling precise differentiation between individuals with and without dementia. The patient is instructed to enumerate all the animals they know for 1 min. Repetitions are not penalized; each animal mentioned is counted only once. A designated cut-off point indicating possible SCD is less than or equal to 10 words. The questionnaire is characterized by its simplicity and rapid application, demonstrating notable sensitivity (74%) and specificity (80%) for cognitive impairment (22).

The rationale behind employing these three tests was to maximize the identification of true positives, thereby enhancing the overall accuracy of the diagnostic process (20–26). The principal aim of this project is to ascertain the incidence of SCD. Each cognitive test evaluates distinct facets of the hippocampus, and their simplicity renders them suitable for administration by illiterate individuals (24). In this study, the utilization of three tests is deemed instrumental in obtaining a comprehensive understanding of cognitive impairment within our population. Past research demonstrates that employing this methodology has resulted in the diagnosis of 90% of individuals referred to neurology services (18, 27). Individuals exhibiting at least one result indicative of SCD on any of the three tests used were subsequently referred to primary care for further assessment, as those with possible SCD following cognitive evaluation were deemed to be at an elevated risk of developing dementia (12).

Additional lifestyle-related protective and/or risk factors, such as these related to cognitive reserve (education level, reading, occupation or board games), vascular risk factors (BMI, AHT, dyslipidaemia, or tobacco consumption) lifestyle factors (physical activity, nutrition and sleep) and depression were evaluated as correlated variables in the questionnaire. In addition to these parameters, the current medication used was documented for subsequent analysis, including the calculation of the anticholinergic burden using the CALS methodology (13).

**Figure 2:**
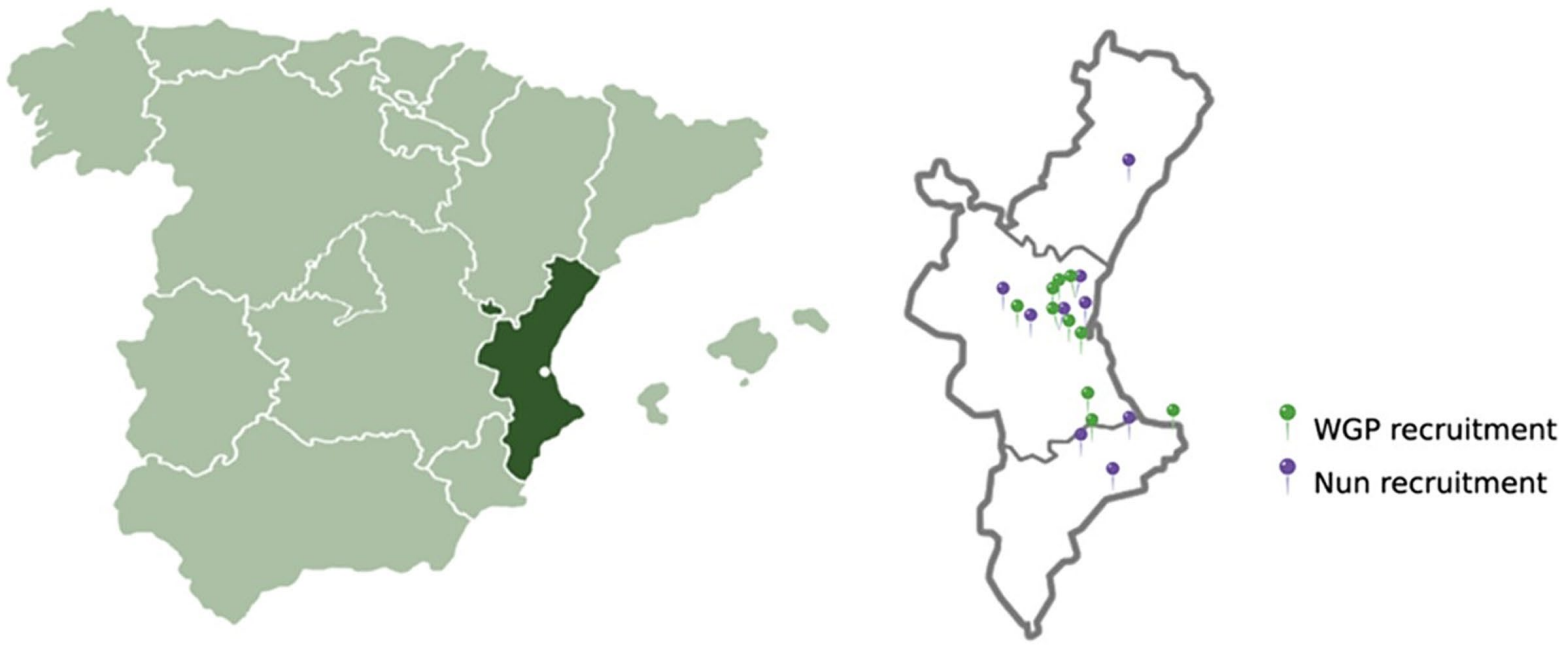
Geolocation of recruitment of study participants. The green pins indicate the pharmacies where the WGPs were recruited, and the purple pins indicate the convents where the nuns were screened.

Occupation was quantified based on an individual’s activity or responsibilities within society. The roles undertaken by nuns within religious congregations were evaluated and correlated with the classification of jobs performed by WGPs (legend Figure 2). In the other hand, the patients were inquired about the number of hours they slept during the day, the frequency of their weekly physical activity, and the duration of each exercise session (hours).

To evaluate and compare nutritional status, in a separate cross-sectional observational study including the same nun population who were the subject of the study, and 180 women over 50 years from the general population of Valencian region (Spain), the same geographical location as the population of nuns. Adherence to the Mediterranean diet was determinate using the PREDIMED questionnaire (MeDAS-14) (28). In addition, the Mini Nutritional Assessment (MNA) questionnaire (29) was used to rate the patients’ risk of malnutrition.

The MeDAS-14 (Mediterranean Diet Adherence Screener) (28) test from PREDIMED is a tool designed to assess adherence to the Mediterranean diet. It consists of 14 questions that evaluate the frequency of consumption of key components of the Mediterranean diet, such as fruits, vegetables, nuts, fish, olive oil, and legumes. It also includes questions about the intake of less healthy food items, such as red meat, butter, and sugary beverages. The test helps to identify dietary patterns and provides a score that reflects how closely an individual’s eating habits align with the Mediterranean diet, which is associated with various health benefits, including reduced risk of cardiovascular diseases and improved overall health.

The MNA (Mini Nutritional Assessment) (29) test is a screening tool for assessing the nutritional status of older adult patients. It evaluates anthropometric measurements (BMI, weight loss, mid-arm, and calf circumferences), general health (mobility, lifestyle, medication use, psychological stress), dietary habits (meal frequency, feeding autonomy, food and fluid intake), and subjective health and nutrition perceptions. The MNA provides a score that classifies individuals as well-nourished, at risk of malnutrition, or malnourished, guiding healthcare providers in developing appropriate nutritional interventions.

### 2.5 Statistical treatment

Data obtained from participants were stored in an Excel worksheet. Subsequent to data collection, the database underwent refinement, and statistical processing was executed using the advanced statistical software RStudio (RRID:SCR_000432) (30).

To examine statistically significant differences between both groups, two-sample t-tests were conducted after testing compliance with the assumptions of normality (Shapiro–Wilk test) and homogeneity of variances (Levene’s test). In case such assumptions were not met, an equivalent non-parametric alternative to the t-test was utilized. In this regard, the Wilcoxon rank-sum method or the Welch two-sample t-test was considered. The significance level was established at 0.05 for all tests. Moreover, additionally, Pearson’s Chi-squared tests were performed to assess relationships among various qualitative factors, and Fisher’s exact test was also employed in the analysis of the corresponding contingency tables, when needed. For the execution of all tests, the “Crosstable” R library was employed to perform the exploratory statistical inference (31).

## 3 Results

### 3.1 Participants and subtle cognitive decline screening

A total of 150 nuns, representing 13 distinct religious orders, and 172 lay women from the general population (WGP) were enrolled in the study. The average age for both nuns and control participants were 76.7 ± 11.7 and 75.7 ± 9.2, respectively, with no statistically significant differences observed (*p*-value >0.05). Subjective memory complaints were reported by more than half of the participants in both groups, with percentages of 58.00% for nuns and 69.19% for lay women. The nuns’ group was further categorized into three subgroups based on their origin: contemplative, caregivers and educational. No significant differences were found among these subgroups, leading us to consider them collectively as a single study group. The geolocation of the recruited patients is shown in Figure 2.

**Figure 3:**
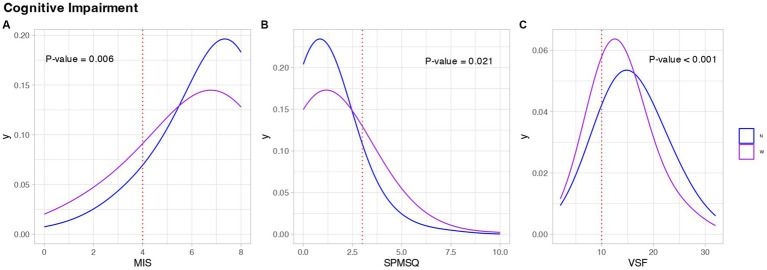
Cognitive assessment of the study population. Each graph refers to the results of **(A)** Memory Impairment Screen (MIS), **(B)** Short Portable Mental Questionnaire (SPMSQ), **(C)** Semantic Verbal Fluency Test (SVF). In the legend, the letter ‘N’ represents the nuns’ group, the letter ‘W’ represents the WGPs. Blue represents Nuns, purple represents WGPs.

The results obtained from SCD screening tests revealed a significantly higher percentage of WGP with outcomes indicative of some form of cognitive decline (36.63% WGP vs. 20.67% of the nun group). This disparity remained significant when analyzing both the cognition tests individually and when combined into a single group (*p*-value <0.05), as illustrated in Figure 3. The density plots visually depict a discernible shift in the curves representing both populations, and the associated *p*-values confirm the presence of significant differences. Specifically, Plot A illustrates a shift of the nun curve towards higher MIS scores compared to the WGP group, while Plot C demonstrates a similar trend for the VSF test. However, Plot B reveals a shift indicating lower scores on the SPMSQ for the WGP group in general.

### 3.2 Cognitive reserve factor

**Figure 4:**
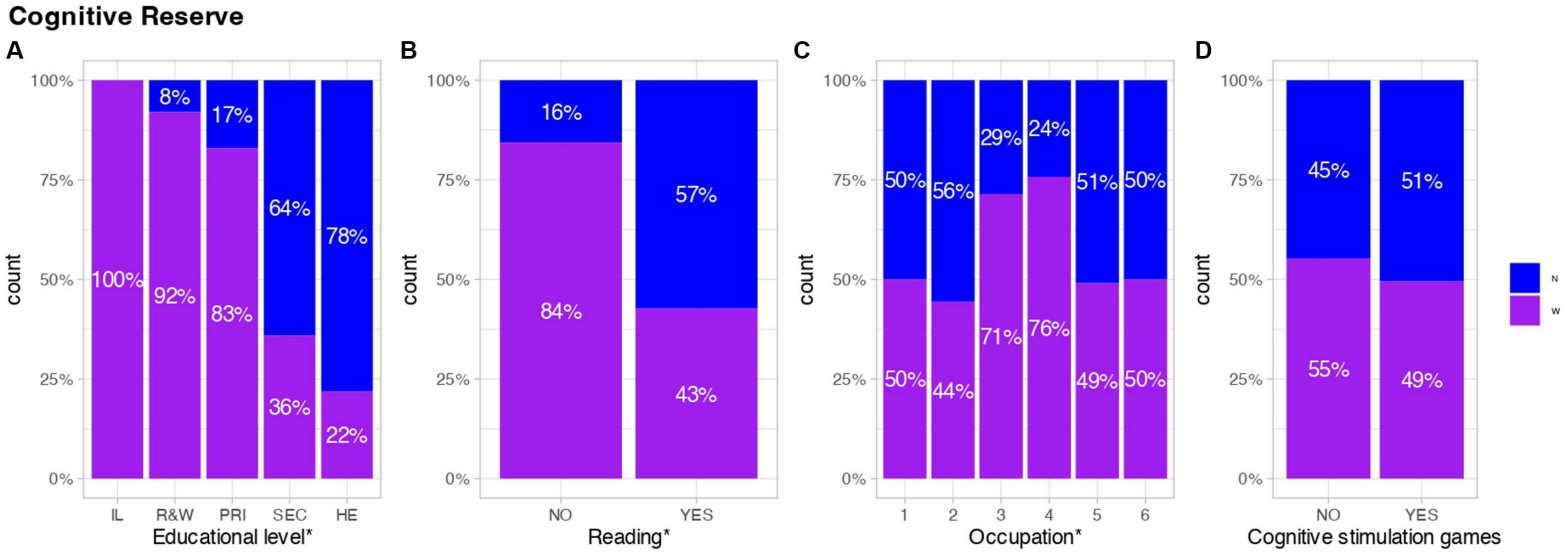
Representation of factors associated with better cognitive reserve. The x-axis in each graph represents: **(A)** IL; illiterate. R&W; Read and write. PRI; primary education. SEC; Secondary education. HE; Higher education. **(B)** This graph refers to reading habits. **(C)** 1; Managers in public administration or companies with more than 10 employees. 2; Managers in companies with less than 10 employees. 3; Skilled non-manual work. 4; Skilled manual work. 5; Semi-skilled manual work. 6; Unskilled manual work. **(D)** This graph shows the practice of cognitive stimulation games. The results are shown cumulatively over the total number of persons with or without any of the assessed factors (18) (pharm). Blue represents Nuns, purple represents WGPs. *Significant differences, (*p*-value <0.05).

Concerning cognitive reserve, various factors including the level of education, reading habits, occupation, and engagement in cognitive stimulation games were investigated. Generally, the nun group exhibited a greater cognitive reserve, as evidenced by their prevalence in the highest positions within the studied areas (Figure 4). Specifically, the nuns demonstrated a higher level of education, with none classified as “illiterate,” and 70% of this group distributed between secondary and higher education (*p*-value <0.001). In contrast, only 22% of all women who completed higher education were part of the WGPs group (see Figure 4A).

Regarding occupation, significant differences were observed in the distribution of individual tasks, with the majority of both groups concentrated in non-skilled jobs, such as cleaning, care for the sick, cooking, etc. However, there were discernible variations in the distribution of more cognitively demanding roles, with only 5.33% of nuns and 4.65% of WGPs engaged in such tasks (*p*-value: 0.0309). Additionally, a distinct pattern emerged in the distribution of nuns across occupational categories, with assignments ranging from congregational management to care and kitchen work. Conversely, individuals from the general population were more evenly distributed in semi-skilled jobs, as depicted in Figure 4C.

No significant differences were observed in the engagement with cognitive stimulation games between the two groups, with over 30% of individuals in each group participating in various cognitive stimulation activities, such as sudoku, crosswords, or puzzles, on a weekly basis (*p*-value >0.05). However, substantial disparities were identified in reading levels (*p*-value <0.001), as depicted in Figures 4B,D.

### 3.3 Cardiovascular risk factor

**Figure 5:**
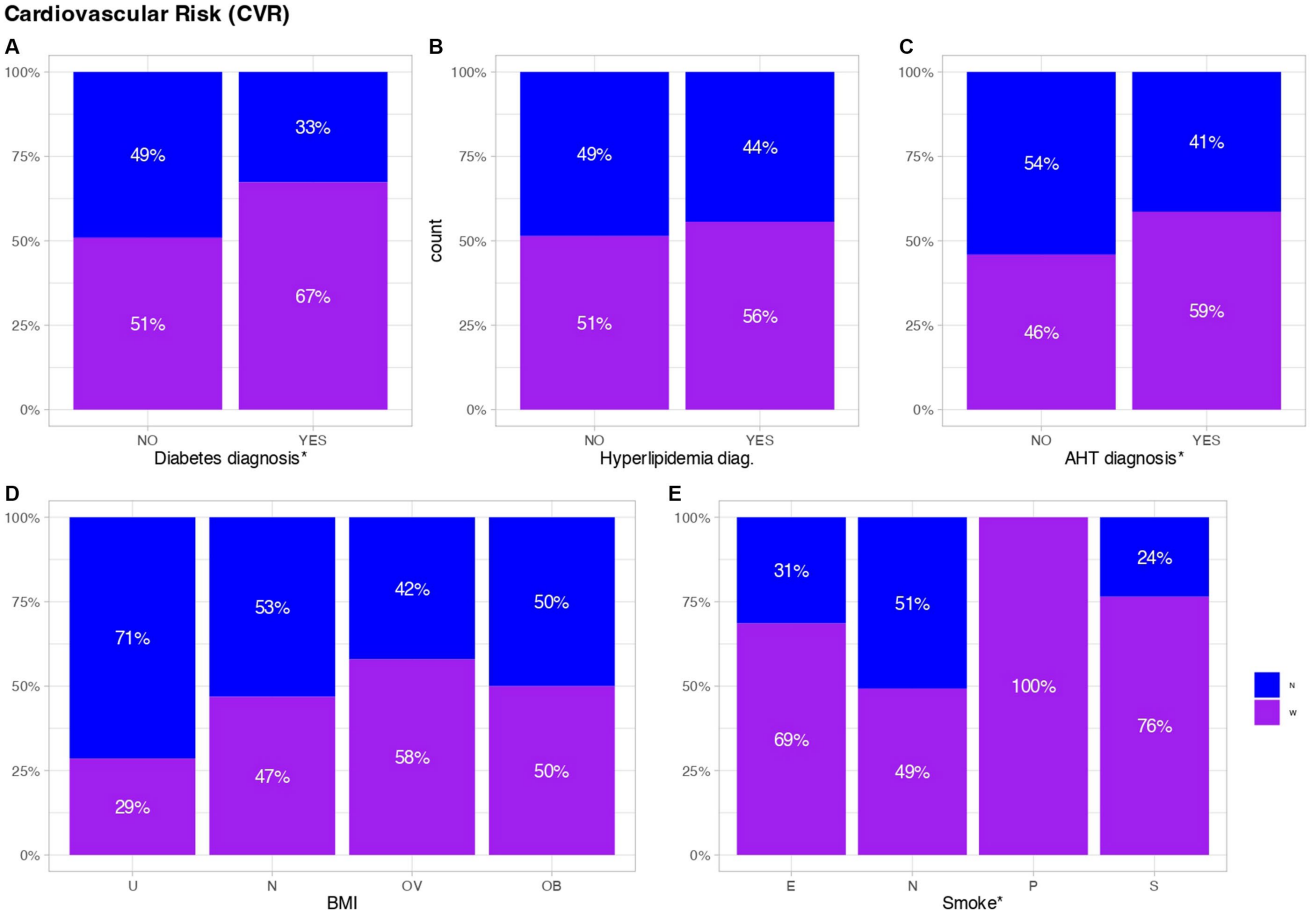
Cardiovascular risk factors. Each graph refers to the results of **(A)** Diabetes diagnosis, **(B)** Hyperlipidemia diagnosis, **(C)** Arterial hypertension (AHT) diagnosis, **(D)** Body Mass Index (BMI), and **(E)** Smoking habits. The bottom of graph **(D)** represents the following: U; Under. N; Normal. OV; Over. OB; Obese. The bottom of graph **(E)** represents the following: E; Ex-smoker. N; Non-smoker. P; Passive smoker. S; Smoker. The results are shown cumulatively over the total number of persons with or without any of the assessed factors. Blue represents Nuns, purple represents WGPs. *Significant differences, (*p*-value <0.05).

On the other hand, factors influencing the progression of cardiovascular risk as a potential risk factor for SCD were investigated. Within the CVR parameters, BMI, the presence of diabetes, AHT or hyperlipidemia, and tobacco consumption in the selected population were examined. No significant differences were observed for BMI or hyperlipemia (*p*-value >0.05). However, significant differences were identified in the diagnosis of AHT (*p*-value: 0.0291), revealing a higher percentage of WGP suffering from this pathology (63.37%), compared to 51.33% in the nun group. Simultaneously, statistically significant differences were found in terms of tobacco consumption (*p*-value: 0.0082), indicating a lower incidence of smoking among nuns (2.67%) compared to the WGP (7.56%), and in the diagnosis of diabetes (*p*-value: 0.033), with a higher percentage of WGP presenting this pathology (19.19% WGP vs. 10.67% Nun) (Figure 5).

### 3.4 Depression and psychopharmaceutical’s consumption

**Figure 6:**
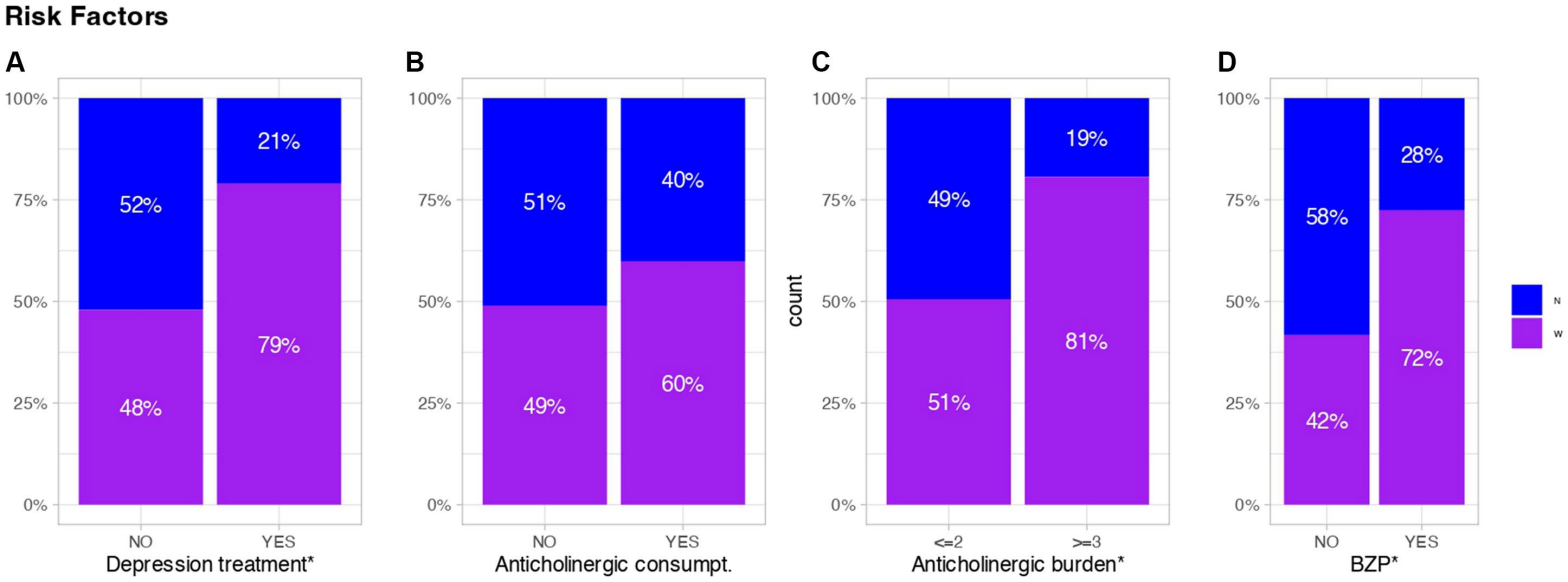
Representative values of depression and treatments affecting cognition. Each graph refers to the results of **(A)** Depression treatment, **(B)** Anticholinergic consumpt, **(C)** Anticholinergic burden and **(D)** Benzodiazepine consumpt. The anticholinergic burden, represented in **(C)**, is considered detrimental when it is ≥3. The results are shown cumulatively over the total number of persons with or without any of the assessed factors. Blue represents Nuns, purple represents WGPs.

Significant disparities in terms of mental health and depression were evident between the two groups. The nun’s group exhibited a lower incidence of depression, with only 7.33% diagnosed compared to 34.33% in the WGP (*p*-value <0.001). Additionally, there was a lower prevalence of benzodiazepine (BZP) consumption in the nun’s group (22.67%) compared to 52.33% in the WGP group (*p*-value <0.001). Moreover, the use of drugs with anticholinergic effects was also lower in the nun’s group (35.33%) compared to the WGP (46.51%), although this difference did not reach statistical significance (*p*-value >0.05). Furthermore, among those who consumed this type of drug, significant differences in anticholinergic burden were observed between the two groups (*p*-value 0.029; Figure 6), with a greater average in WGP group.

### 3.5 Lifestyle factors

The study assessed factors pertaining to the participants’ lifestyles, revealing no statistically significant variance in sleep duration between both groups (*p*-value: 0.3574). However, significant differences were observed in physical activity levels, with an average of 5.2 ± 3.2 h per week for the WGP, compared to 7.5 ± 5.2 h per week among the nuns (*p*-value <0.001), as detailed in Table 1.

**Table 1:** Lifestyle factors.

Factor	Variable	WGP	Nun	*p*-value
Sleep	Min / Max	3.5/11.5	4.0 / 12.0	*p*-value^a^: 0.3574
	Med [IQR]	7.0 [6.0;8.0]	7.0 [6.0;7.0]	
	Mean (std)	7.0 (1.6)	6.8 (1.1)	
Physical activity	Min / Max	1.2 / 17.5	1.2 / 35.0	***p*-value**^**a**^ < 0.001^*^
	Med [IQR]	4.5 [3.0;7.0]	7.0 [3.5;7.0]	
	Mean (std)	5.2 (3.2)	7.5 (5.2)	

a Wilcoxon rank sum test.

^*^Significant differences, (*p*-value < 0.05).

In the parallel study, we assessed the nutritional status and adherence to the Mediterranean diet in the group of nuns and in the control group of women. We found no significant differences in nutritional status (*p*-value: 0.1127) or adherence to the Mediterranean diet (*p*-value: 0.8997) between the two groups.

### 3.6 Effects on subtle cognitive decline

Following the identification of similarities and differences between the two groups, we opted to assess and compare the same parameters among individuals exhibiting SCD.

The initial observation pertains to significant differences in the age of the two groups (*p*-value: 0.0042), where in WGPs exhibited results indicative of SCD at an earlier age compared to the nuns (78.4 ± 8 and 83.4 ± 7.2, respectively). In this context, there were no significant differences in the cognitive tests employed (*p*-value >0.05).

Regarding the cognitive reserve, we observed that the educational level and the reading frequency continued to show significant differences between both groups (*p*-value <0.01), with the nuns’ group having a higher educational level but also a higher percentage of members positive for SCD who read frequently (*p*-value <0.001; Table 2).

**Table 2:** Protective factor, cognitive reserve, in cognitively impaired individuals.

Factor	Variable	SCD positive		*p*-value
		WGP	Nun	
Education level	Illiterate	4 (6.35%)	0 (0%)	*p*-value^a^ < 0.001^*^
	Read and write	25 (39.68%)	3 (9.68%)	
	Primary education	19 (30.16%)	6 (19.35%)	
	Secondary education	10 (15.87%)	11 (35.48%)	
	Higher education	5 (7.94%)	11 (35.48%)	
Reading	No	36 (57.14%)	7 (22.58%)	*p*-value^b^ < 0.001^*^
	Yes	27 (42.86%)	24 (77.42%)	
Occupation	Managers in companies with >10 employees	1 (1.59%)	1 (3.23%)	*p*-value^a^: 0.435
	Managers in companies with <10 employees	5 (7.94%)	1 (3.23%)	
	Skilled non-manual work	3 (4.76%)	1 (3.23%)	
	Skilled manual labor	9 (14.29%)	1 (3.23%)	
	Semi-skilled manual work	10 (15.87%)	4 (12.90%)	
	Unskilled manual work	35 (55.56%)	23 (74.19%)	
Cognitive stimulation games	No	45 (71.43%)	20 (64.52%)	*p*-value^b^: 0.495
	Yes	18 (28.57%)	11 (35.48%)	

a Fisher’s Exact test for count data.

b Pearson’s Chi-squared test.

^*^Significant differences, (*p*-value < 0.05).

Thirdly, we examined the factors influencing CVR. In contrast to the observations made when comparing both groups regardless of cognitive status, significant differences in AHT and smoking were no longer evident in the SCD subpopulation (*p*-value >0.05) (Table 3).

**Table 3:** Risk factors in cognitively impaired individuals.

Factor	Variable	SCD positive		*p*-value
		WGP	Nun	
BMI	underweight	0 (0%)	2 (6.45%)	*p*-value^b^: 0.068
	Normal	17 (26.98%)	12 (38.71%)	
	Overweight	45 (71.43%)	16 (51.61%)	
	Obese	1 (1.59%)	1 (3.23%)	
AHT diagnosis	No	19 (30.16%)	12 (38.71%)	*p*-value^a^: 0.321
	Yes	45 (71.43%)	19 (61.29%)	
Diabetes diagnosis	No	53 (84.13%)	27 (87.10%)	*p*-value^b^: 1.000
	Yes	10 (15.87%)	4 (12.90%)	
Hyperlipidaemia diagnosis	No	34 (53.97%)	20 (64.52%)	*p*-value^a^: 0.321
	Yes	29 (46.03%)	11 (35.48%)	
Smoke	Ex- smoker	8 (12.70%)	0 (0%)	*p*-value^b^: 0.107
	Passive smoker	1 (1.69%)	0 (0%)	
	Smoker	2 (3.17%)	1 (3.23%)	
	Non- smoker	52 (82.54%)	30 (96.77%)	

a Pearson’s Chi-squared test.

b Fisher’s Exact test for count data.

Factors such as depression or pharmacological treatments that may impact mental health were also subjected to comparison (Table 4). The presence of depression remains significantly different between the two groups, with 36.68% of SCD-positive WGPs versus 12.90% of SCD-positive nuns (*p*-value = 0.0082). Concerning the use of BZP, there were also significant differences between both groups (*p*-value = 0.0484). Conversely, anticholinergic consumption and burden were no longer significantly different between both SCD groups (Table 4).

**Table 4:** Depression and pharmacological treatments that affect mental health.

Factor	Variable	SCD positive		*p*-value
		WGP	Nun	
Depression	No	38 (60.32%)	27 (87.10%)	***p*-value** ^**a**^: 0.008^*^
	Yes	25 (39.68%)	4 (12.90%)	
Benzodiazepine consumption	No	25 (39.68%)	19 (61.29%)	***p*-value** ^**a**^: 0.048^*^
	Yes	38 (60.32%)	12 (38.71%)	
Anticholinergic consumption	No	32 (50.79%)	16 (51.61%)	*p*-value^a^: 0.940
	Yes	31 (49.21%)	15 (48.39%)	
Anticholinergic Burden CALS	Min / Max	1.0 / 5.0	1.0 / 5.0	*p*-value^b^: 0.106
	Med [IQR]	1.0 [1.0;3.0]	1.0 [1.0;2.0]	
	Mean (std)	1.9 (1.1)	1.6 (0.9)	

a Pearson’s Chi squared test.

b Wilcoxon rank sum test.

^*^Significant differences, (*p*-value < 0.05).

Lastly, concerning lifestyle factors, sleep continues to exhibit no notable variances between the two populations (*p*-value: 0.7879). Conversely, while physical activity had previously demonstrated significant distinctions between WGPs and nuns, its significance diminishes when examining SCD populations (*p*-value: 0.1063).

## 4 Discussion

Consistent with findings from analogous studies (15–17) and considering the similarities in age between the groups, the prevalence of SCD in the WGP was notably higher than in the nun population. Furthermore, the WGP demonstrated the onset of SCD at an earlier age compared to the nun group. These variations in SCD prevalence may be explained by disparities in specific risk and/or protective factors. Nuns exhibited significantly lower values for risk factors, including AHT, tobacco consumption, depression, and diabetes, as well as anticholinergic burden and benzodiazepine consumption. Conversely, they manifested significantly higher values for protective factors such as educational level and reading habits.

Another factor influencing SCD is loneliness and social isolation, which is evidently distinct in the two study populations. This factor may be present in the WPG but is consistently absent in the nuns group. While social isolation and loneliness are recognized as primary risk factors for SCD in older adult individuals (9), this factor diminishes within the nun’s group due to their communal living in religious communities. Active participation in social activities and the sustained maintenance of social connections have been correlated with enhanced cognitive health (32).

Moreover, it has been demonstrated that religiosity, both organized and non-organized, impacts depression outcomes and potentially influences cognitive functioning (33, 34). The relationship between religion and mental health has been explored in various studies (35–37), suggesting that religion serves as a significant means of finding meaning and purpose in life, contributing to a sense of wellbeing. A total of 454 recent studies (344 of which were published after 2000) highlight how religion/spirituality proves beneficial in assisting individuals in coping with diverse stressful situations, symptoms, natural disasters, illnesses, and various adverse life circumstances, including bereavement and end-of-life conditions. Furthermore, several studies indicate that engaging in inner religious and spiritual experiences (e.g., prayer) is advantageous for mental health and contributes to a quicker recovery from illness (38).

The Nun Study (15) highlighted that the structured lives of nuns, characterized by lower stress levels and increased engagement in cognitive and social activities, may contribute to their relatively better cognitive health.

The most noteworthy data of significance, when comparing the same parameters between the two groups with results compatible with SCD, revolves around a substantial disparity in age between the population groups manifesting symptoms indicative of SCD. In this context, WGP exhibit an earlier onset of SCD manifestations compared to the Nun’s group. The delayed onset of SCD in the Nuns group could be explained by the significant difference that remains, in this second comparison, in some factors that are more favorable to nuns. These factors include a higher level of education, greater engagement in reading, lower levels of depression, and reduced benzodiazepine consumption.

### 4.1 Cognitive reserve factor

Cognitive reserve, denoting the brain’s capacity to alleviate age-related changes and pathological processes, constitutes a complex construct shaped by a confluence of genetic, environmental, and lifestyle factors (39). Although education is universally acknowledged as a pivotal environmental factor in the cultivation of cognitive reserve, it is imperative to recognize the crucial roles played by other factors, including occupation and involvement in intellectually stimulating activities, in the prevention or postponement of cognitive decline (40, 41).

The positive impact of education on cognitive reserve has long been established (9). Higher educational attainment is correlated with enhanced cognitive abilities and a decreased risk of SCD (42). The acquisition of cognitive knowledge and skills through formal education can augment neural connections and promote cognitive flexibility (40), both of which constitute fundamental components of cognitive reserve. In our study, we noted a higher educational level among nuns compared to the WGP. When scrutinizing the educational levels within the populations exhibiting symptoms consistent with SCD, we observed a consistent distribution with significantly higher educational levels in the nun group, while lower educational levels were observed among the WGP (Table 2).

Occupation also plays a substantial role in the development of cognitive reserve. Occupations that involve intellectual demands, necessitating problem-solving, critical thinking, and continuous learning, have consistently demonstrated a correlation with higher cognitive reserve and a diminished risk of cognitive decline. Engaging in occupations that entail complex tasks and intellectual challenges offers ongoing opportunities for cognitive stimulation, thereby contributing to the establishment and maintenance of cognitive reserve throughout an individual’s lifespan (3, 43).

In our initial comparison, we noted that the group of nuns exhibited significantly higher levels of education and lower levels of occupation compared to WGP. This significant difference persists only in the realm of educational levels when comparing populations with SCD within each sample. However, the distribution of educational levels in relation to the occurrence of SCD in the nun group is inverse (Table 2). This phenomenon may be attributed to the fact that within the convent, the occupation of each nun is not correlated with their educational level. It is accurate to state that the nuns predominantly engage in more manual activities within the congregation. However, it should be noted that this is a population that does not retire; when they can no longer perform one activity, they transition to another within the congregation, underscoring the enduring importance of occupation throughout life for the prevention of SCD.

Another factor, engagement in reading, was notably higher for the nuns compared to the WGP (Figure 3). However, when scrutinizing individuals displaying symptoms of SCD, we observed that engagement in reading remains significantly higher in the nun group. This factor could play a crucial role in preserving memory and cognitive function for nuns who develop SCD later in life compared to the WGP (Table 2). This underscores the importance of reading significance of reading as a pivotal activity for sustaining cognitive health (9, 39).

Moreover, it is important to acknowledge that cognitive activity alone may not provide absolute protection against cognitive decline or dementia. Nevertheless, it can serve as a pivotal component within a holistic lifestyle approach aimed at cultivating optimal brain health. Furthermore, the integration of complementary lifestyle factors, such as regular physical exercise and active social engagement, has demonstrated potential benefits in enhancing cognitive well-being (44).

### 4.2 Cardiovascular risk factor

There is evidence to suggest that CVR factors can impact cognitive function and increase the risk of SCD. However, the concept of cognitive reserve posits that individuals with higher cognitive reserve may be better equipped to manage the effects of cardiovascular risk factors on cognitive function (39).

Numerous factors can influence CVR including age, gender, AHT, hyperlipidaemia, diabetes, IBM and smoking, among others (45–48). It is crucial to effectively manage these risk factors through lifestyle modifications and, in certain cases, medication, to mitigate the likelihood of developing cardiovascular disease. The presence of CVR factors becomes evident, subsequently influencing the progression of SCD (49). It is noteworthy that the nuns manifested significantly lower levels of AHT, diabetes, and tobacco consumption (*p*-value <0.05) (Figure 3). Importantly, tobacco consumption, AHT and diabetes assume a substantial role as significant risk factors for cardiovascular disease. Additionally, smoking further augments blood pressure and heart rate, compounding the risk of cardiovascular disease (48). Nevertheless, following the identification of SCD symptoms, none of these factors demonstrated statistically significant distinctions between the groups (*p*-value >0.05) (Table 3).

### 4.3 Depression and psychopharmaceutical’s consumption

SCD associated with depression is characterized by a decline in memory, attention, and decision-making abilities, which may persist even after the remission of depressive symptoms (9, 50). This impairment is linked to abnormalities in brain function and structure, including disruptions in the neurotransmitter system and a decrease in volume in specific brain regions. Initially, the two groups demonstrated significant differences in depression, BZP consumption, and anticholinergic burden (*p*-value <0.05). However, when cognitive decline is present, only depression and BZP consumption maintain their significance.

Religiousness serves as a protective factor against depression and contributes to its recovery (51). Furthermore, some studies suggest that religious commitment may be associated with better cognitive functioning (52), which could potentially explain the lower occurrence of depression in the nun group. Research findings indicate that individuals with a religious orientation exhibit effective coping mechanisms in stressful situations and are more likely to display resilience when confronted with adversity (53). These results provide justification for the observed differences between the two groups.

Additionally, the group of nuns, are all involved in social work, whether it is teaching, caring for the sick or praying for others. It has been described that social engagement can reduce the risk of depressive symptoms (54).

On the other hand, while BZPs may be effective in controlling anxiety and sleep disorders in the short term, prolonged use can lead to cognitive problems (55, 56). BZPs can have similar effects to anticholinergic drugs and can overlap with drug-associated SCD (13, 57). They can increase the risk of falls and contribute to cognitive decline in older adults, justifying the significant differences found between the two populations (Table 3). Additionally, benzodiazepines can lead to habit formation and dependence or addiction, especially with prolonged use.

### 4.4 Lifestyle factors

It is noteworthy that regular physical activity, nutritional supplementation, and sleep habits significantly impact cognitive function and overall physical and mental health. Understanding these lifestyle factors’ roles is crucial for a comprehensive view of health (58–62).

Significant relationships have been observed between inflammatory markers and sleep disturbances, with sleep playing a crucial role in regulating the immune system and disruptions linked to increased inflammation (58). Lack of sleep is associated with various health consequences, including impaired cognitive functions such as thinking and memory, and prolonged wakefulness can detrimentally affect cognitive performance. These findings underscore the importance of adequate sleep for maintaining cognitive abilities.

Furthermore, a sedentary lifestyle is strongly associated with elevated levels of cytokines involved in regulatory and inflammatory processes (58). Other studies have found that higher sedentary time and lower physical activity levels are linked to higher C-reactive protein levels, an inflammation marker. Replacing sedentary time with moderate-to-vigorous physical activity significantly reduces C-reactive protein levels (61). Also, Dominguez et al. argue that regular physical activity is highlighted as a critical factor in maintaining cognitive function and preventing dementia (62).

The study assessed various lifestyle factors among participants, revealing no significant difference in sleep duration between the two groups (*p*-value: 0.3574). However, a significant difference in physical activity levels was observed, with the WGP averaging 5.2 ± 3.2 h per week compared to 7.5 ± 5.2 h per week among the nuns (*p*-value <0.001), as detailed in Table 1.

These findings are consistent with other studies that have found a positive relationship between education level and physical activity, showing that people with more education are more likely to be physically active (59). Moreover, is described than behavioral intervention such as increasing moderate to vigorous exercise has been detected to exhibit approximate antidepressant effects (60). The population of nuns studied has a higher level of education, is more physically active and has a lower incidence of depression. This increased physical activity among the nuns may have a positive effect in delaying the onset of cognitive decline.

In a parallel study was examined the nutritional status and adherence to the Mediterranean diet among the population of nuns who were the subject of the study, and a control group of women. This assessment found no significant differences in nutritional status (*p*-value: 0.1127) or adherence to the Mediterranean diet (*p*-value: 0.8997), which was expected due to the geographical proximity of both populations.

The potential implications for using diet and exercise strategies to modulate inflammatory responses have far-reaching implications, with promising avenues for preventive and therapeutic interventions in the field of chronic inflammatory diseases. The combination of a healthy diet and regular physical activity has a synergistic effect on cognitive health. Both factors work together to enhance brain function, reduce oxidative stress, and improve overall physical health, which in turn supports cognitive resilience (62).

### 4.5 Risk and protective factors on subtle cognitive decline

SCD serves as an intermediary phase that bridges the cognitive changes associated with normal aging and the characteristic manifestations found in dementia. The clinical signs of this pathology become evident up to two decades prior to the detectability of the disease. Understanding relevant risk and protective factors, their magnitudes, and their interplay is crucial for maintaining a high quality of life over an extended period. To our knowledge, this study represents the first European investigation to systematically assess risk factors and protective factors for SCD, utilizing a homogeneous cohort of women from religious orders as the study population. The selection of this group stems from their remarkable homogeneity, owing to their long-term shared lifestyle, thereby minimizing sample variability.

The findings of this study provide valuable insights into the risk and protective factors associated with SCD in a homogeneous cohort. The observed associations between specific factors and SCD progression contribute to the existing knowledge regarding the aetiology and pathogenesis of SCD. These findings have significant implications for the development of targeted interventions and strategies to delay or mitigate the cognitive decline observed during this transitional phase. In summary, nuns may have a lower risk of cognitive decline and dementia compared to the general population but when SCD appears, it is quite similar regarding the presence of risk factors to that seen in the general population. This lower risk for cognitive deterioration may be partly attributed to a combination of lifestyle-related factors and social engagement. Nuns often possess greater cognitive reserve and engage in intellectually stimulating activities, such as reading and socializing, on a regular basis. This can help promote cognitive function and delay the onset of cognitive decline. Additionally, nuns tend to lead healthy lifestyles, incorporating regular exercise and a balanced diet, resulting in lower cardiovascular risk that further support cognitive health. This study has shown a lower incidence of cognitive decline among nuns, and a delayed age of onset, and highlights the importance of individuals taking steps to promote cognitive health and reduce the risk of cognitive decline, irrespective of their lifestyle or genetic factors. This may include engaging in regular physical exercise, a healthy diet, maintaining an appropriate weight, staying mentally and socially active, managing chronic conditions such as diabetes and hypertension, and avoiding or minimizing alcohol, tobacco, and drugs with anticholinergic burden consumption.

This study aims to highlight the importance of risk or preventive factors for SCD. Previous studies addressed an interprofessional collaboration between community pharmacists, physician, and neurologists for mass screening, detection of population with subjective memory complaint and assessment of preventive and risk factors for SCD in each individual. As a result of this interdisciplinary collaboration, 90% of patients detected as possible SCD were subsequently diagnosed with dementia (18). This highlights the importance of a multidisciplinary approach to patient care.

## 5 Limitations

The present study encountered certain limitations in its execution. Firstly, essential information regarding participants, such as musical training, parents’ educational level, completed formal courses, or the number of languages spoken in a conversation, was not accessible. This limitation hindered our ability to make a precise estimation of cognitive reserve. Secondly, the degree of loneliness in women from the general population was not recorded, preventing a comparison with religious women. Additionally, the level of religiosity in both populations was not assessed. Furthermore, according to the socio-political situation in Spain and Europe during the 1930s and 1940s, this sample of women could not be compared with men within the same age range. Our study research area is the prevention of neurodegenerative diseases, but we found interesting the study from a socio-political and cultural point of view for another project.

## Data availability statement

The raw data supporting the conclusions of this article will be made available by the authors, without undue reservation.

## Ethics statement

The studies involving humans were approved by Institutional Review Board (IRB) at the CEU Cardenal Herrera University (CEEI21/201, approval date: 16 July 2021). The studies were conducted in accordance with the local legislation and institutional requirements. The participants provided their written informed consent to participate in this study.

## Author contributions

TL: Conceptualization, Investigation, Methodology, Writing – original draft. LM: Conceptualization, Investigation, Methodology, Supervision, Validation, Writing – review & editing. JP: Conceptualization, Data curation, Formal analysis, Software, Writing – review & editing. JP-T: Conceptualization, Writing – review & editing. HR: Conceptualization, Investigation, Methodology, Writing – review & editing. VV: Conceptualization, Funding acquisition, Investigation, Methodology, Project administration, Supervision, Validation, Writing – review & editing.
